# Trace Metal Detection in Aqueous Reservoirs Using Stilbene Intercalated Layered Rare-Earth Hydroxide Tablets

**DOI:** 10.1155/2020/9712872

**Published:** 2020-04-12

**Authors:** Solomon Omwoma

**Affiliations:** Jaramogi Oginga Odinga University of Science and Technology, Box, 210-40601 Bondo, Kenya

## Abstract

Contamination of aquatic reservoirs with metal ions is a slow gradual process that is not easy to detect. Consequences of the metal ions, especially the ones with high atomic numbers (heavy metals) at high concentrations, are severe and irreversible in aquatic reservoirs. As such, early detection mechanisms, especially at trace concentration, are essential for mitigation measures. In this work, a new, robust, and effective tool for trace metal detection and monitoring in aqueous solutions has been developed. Tablets (1 mm thick and similar to medicinal tablets) were manufactured from a powder comprising stilbene intercalated into gallery spaces of lanthanide-containing layered double hydroxides. The tablets were placed in a water column having different concentrations of Pb^2+^ and Cu^2+^ ions, and the water was allowed to flow for 45 minutes at a flow rate of 100 ml/s. Thereafter, the tablets were dried and made to powder, and their phosphorescence was measured. The gradual stilbene phosphorescence turnoff in the tablets from various concentrations of metal ions was correlated with sorption amounts. The tablets were able to detect effectively metal ions (up to Pb^2+^ 1.0 mmol/L and Cu^2+^ 5.0 mmol/L) in the aqueous media. As such, the concentrations of Pb^2+^ and Cu^2+^ ions at trace levels were determined in the test solutions. This method provides a real-time metal ion analysis and does not involve sampling of water samples for analysis in the laboratory.

## 1. Introduction

Loading of heavy metals into aquatic systems such as rivers, swamps, and lakes is a slow and accumulative process that can easily go undetected until the toxicity levels are surpassed [1]. In an effort to prevent aquatic system contaminations, environmental monitoring bodies map possible pollutant sources and perform point source assessment on regular basis [2]. However, the large number of samples required for analysis per day limits such efforts especially in developing economies. In addition, the requirements that the management of the point source must be informed before carrying out such assessment activities [2] may also interfere with the results of such a study. Point source heavy metal evaluation in aquatic systems is therefore a challenge to environmental monitoring bodies.

Sampling water for heavy metal analysis is a tedious, expensive, and heavy exercise that might affect sample population. Any efforts that can help in removing the burden of water sampling for heavy metal analysis can go a long way in improving the data obtained for a particular population. This is particularly the case as the burden of water sampling is removed and the researcher can collect as many samples as possible.

Previously, the diverse effects and transport mechanisms associated with heavy metal ions such as Pb^2+^ and Cu^2+^ from point sources have been reported [3, 4]. Among other diverse effects, their impact on aquatic organisms, including fish and sea foods, greatly affects trade and human health [5, 6]. In all circumstances, the impacts are detected at very critical stages and they are irreversible [3, 5, 6]. Therefore, the need to detect point source contamination earlier enough is very important both to humans and aquatic systems.

In the current work, a tool is developed that is able to detect lead and copper ions in aqueous solutions at trace levels. The tool which is a tablet that is made similar to medicinal tablets is synthesized from stilbene (4, 4-bis (2-sulfonatostyryl) biphenyl: [C_28_H_20_O_6_S_2_]^2−^) intercalated within gallery spaces of lanthanide-containing layered double hydroxides.

Intercalation of stilbene, an anionic organic compound, into layered double hydroxides (LDHs) has been reported and fully characterized by Yan et al. [7]. Furthermore, effective heavy metal sorption by organic intercalated LDHs has also been demonstrated [8, 9]. It has also been found that lanthanide luminescence can be stabilized by organic compounds through the antenna effect [10, 11]. However, presence of heavy metals in such a sample will have a turn off/on effect on the phosphorescence of the organic ligand [12–14]. It is upon the above background that the new environmental monitoring tool for heavy metals has been developed.

Previously, apart from conventional analytical techniques, similar detection mechanisms have been reported for biosensors [15]. Biosensors are analytical tools comprising immobilized biological material in contact with a compatible transducer to convert biological signals into measurable electrical signals. There exists biosensors to detect Cd [16], Zn [17], Hg [18], Cu [19], Ni [20], and Pb [21]. Biosensors have high specificity, are small in size, and respond rapidly to metal concentrations leading to their applications in ecological, monitoring, clinical, and nutritional studies [22, 23]. Nevertheless, inhibition-based biosensors experience insufficient selectivity due to simultaneous inhibition of enzymes by some metals [15]. Other biosensors exhibit interference from contaminants leading to low response, hence inefficiency. Recently, Tian et al. [24] developed a fluorescence “turn on” sensor for Pb^2+^ using carbon nanodots immobilized in spherical polyelectrolyte brushes. They report a great achievement, and it is upon their efforts that the current sensor is developed.

The ability of metal ions to be absorbed by organic materials has also been demonstrated by various researchers including [25–28]. It is upon such background that stilbene, an organic compound, was chosen for metal adsorption. The novelty of our findings lies in the ability of adsorbed metal ions to turn off the phosphorescence of stilbene. The turnoff is gradual and correlates perfectly with metal concentrations even at trace levels.

In the new developed method, the tablets are able to sorb the heavy metals from water in real-time analysis. In conventional analytical techniques, the analyst has to sample water, whereas in the developed technique, the tablets are simply lounged at the desired site.

## 2. Methodology

### 2.1. Chemicals

All the chemicals used in the synthesis of the tablets were purchased from Alfa Aesar and used without further purification unless otherwise indicated.

### 2.2. Preparation of Stilbene Intercalated Lanthanide-Containing LDHs

Lanthanide-containing LDHs (Dy_2_(OH)_5_(C_28_H_20_O_6_S_2_)_0.5_.nH_2_O; Gd_2_(OH)_5_(C_28_H_20_O_6_S_2_)_0.5_.nH_2_O; Nd_2_(OH)_5_(C_28_H_20_O_6_S_2_)_0.5_.nH_2_O; Sm_2_(OH)_5_(C_28_H_20_O_6_S_2_)_0.5_.nH_2_O; Yb_2_(OH)_5_(C_28_H_20_O_6_S_2_)_0.5_.nH_2_O; Er_2_(OH)_5_(C_28_H_20_O_6_S_2_)_0.5_.nH_2_O; Tb_2_(OH)_5_(C_28_H_20_O_6_S_2_)_0.5_.nH_2_O; Eu_2_(OH)_5_(C_28_H_20_O_6_S_2_)_0.5_.nH_2_O) were synthesized according to literature methods [29, 30]. A tablet was made by spreading 0.5 mg of the active compound powder into a pressing machine, 5 mg of a filler, that is, phosphorescent/luminescent inactive which is added (in this case CaCO_3_), and finally 0.5 mg of the active ingredient spread on top before being compressed into a tablet. The resultant tablet, 12 mm wide and 1 mm thick, was used in heavy metal sorption experiments.

### 2.3. Tablet Analysis

The method used previously in analyzing lanthanide-containing LDHs [31] was used to analyze the present tablets. The method is described briefly in the supporting information.

### 2.4. Metal Detection Experiments

The tablets were placed in a water column having different concentrations of Pb^2+^ and Cu^2+^ ions, and the water was allowed to flow for 45 minutes at a flow rate of 100 ml/s. This was done in order to mimic field environmental conditions. Thereafter, the tablets were dried and made into powder and their phosphorescence was measured. Subsequently, the powder samples were digested using an aqua regia (HCl : HNO_3_ = 3 : 1) solution, and the concentration of specific heavy metals was also determined using inductively coupled plasma mass spectrometry (ICP-MS).

## 3. Results and Discussion

### 3.1. Characterization of the Tablets

**Figure 1:**
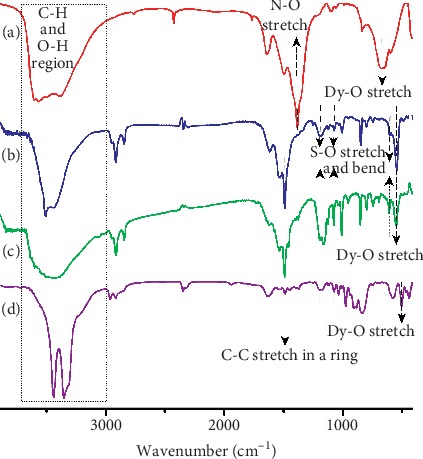
FTIR patterns of (a) Dy_2_(OH)_5_(H_2_O)_n_NO_3_.nH_2_O, (b) Dy_2_(OH)_5_ (C_28_H_20_O_6_S_2_)_0.5_.nH_2_O, (c) Dy_2_(OH)_5_(C_28_H_20_O_6_S_2_)_0.5_@Pb.nH_2_O, and (d) Dy_2_ (OH)_5_(C_28_H_20_O_6_S_2_)_0.5_@Cu.nH_2_O.

In this work, we present results for tablets made from Dy_2_(OH)_5_(C_28_H_20_O_6_S_2_)_0.5_.nH_2_O due to their outstanding performance in detection of heavy metals. Firstly, Dy_2_(OH)_5_(H_2_O)_n_NO_3_.nH_2_O was synthesized before the nitrate was exchanged with stilbene. The FTIR spectra show that the free nitrate anions in Dy_2_(OH)_5_(H_2_O)_n_NO_3_.nH_2_O, with an asymmetric stretching mode at 1396 (D_3h_) cm^−1^ (Figure 1(a)), are completely replaced with stilbene anions, C_28_H_20_O_6_S_2_^2−^, to get the sorbent starting material, Dy-Stb (Figure 1(b)). The asymmetric stretching bands for the intercalated stilbene are recorded at 1172 (*v*_3_), 1072 (*v*_1_), and 650 (*v*_4_) cm^−1^ corresponding to two asymmetric stretches and one asymmetric bend, respectively, for the sulfite molecule on stilbene. [32, 33] The same sulfite vibrations can be located in the spectra of sorbed materials, Dy-Stb@Pb and Dy-Stb@Cu, although extra peaks are noted (Figure 1(c) and 1(d)).

**Figure 2:**
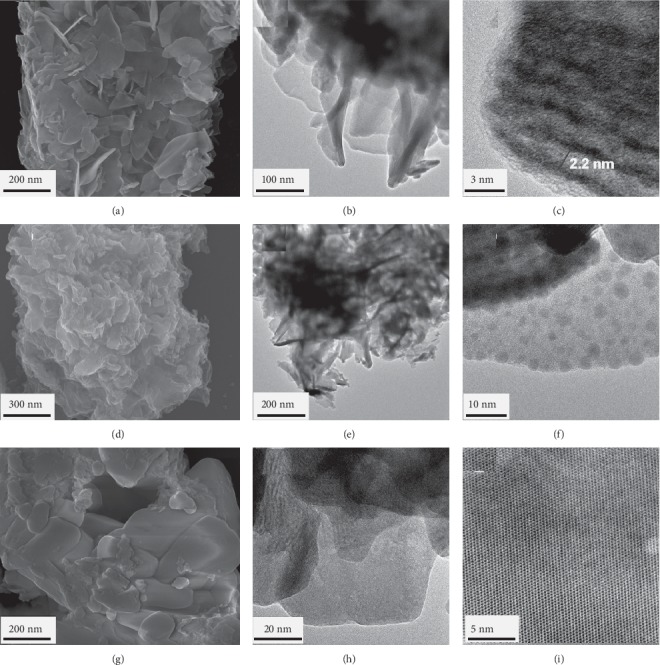
SEM image (first column) and HRTEM images (second and third columns) for Dy_2_(OH)_5_(C_28_H_20_O_6_S_2_)_0.5_.nH_2_O (a–c), Dy_2_(OH)_5_ (C_28_H_20_O_6_S_2_)_0.5_@Cu.nH_2_O (d–f), and Dy_2_(OH)_5_ (C_28_H_20_O_6_S_2_)_0.5_@Pb.nH_2_O (g–i).

**Figure 3:**
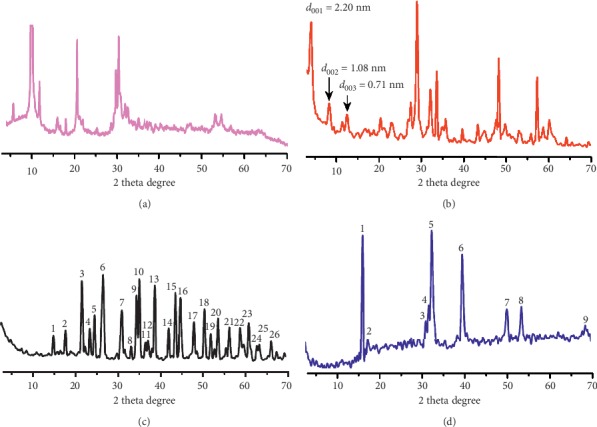
XRD patterns of (a) Dy_2_(OH)_5_NO_3_.nH_2_O, (b) Dy_2_(OH)_5_(C_28_H_20_O_6_S_2_)_0.5_.nH_2_O, (c) Dy_2_(OH)_5_(C_28_H_20_O_6_S_2_)_0.5_@Pb.nH_2_O, and (d) Dy_2_(OH)_5_(C_28_H_20_ O_6_S_2_)_0.5_@Cu.nH_2_O.

The SEM images of the starting material in sorption experiments, Dy-Stb, exhibit plate-like morphology that differs from the amorphous (Figure 2(e)) and block-like (Figure 2(i)) morphology exhibited by the final sorbed samples. HRTEM images also show similar characteristics of morphological differences with the starting material exhibiting distinct plate-like morphology arranged in layers and having a basal spacing of approximately 2.2 nm (Figure 2(a)–2(c)). This basal spacing is in good agreement with the basal spacing found from powder X-ray diffraction (PXRD) measurements in Figure 3(b) which is *d*_001_ = 2.17 nm. The sorbed materials, however, do not exhibit such layered characteristics as seen in SEM, HRTEM, and PXRD measurements (Figures 2 and 3).

The HRTEM images in higher resolution indicate the final sorbed material, Dy-Stb@Pb, to be highly crystalline while Dy-Stb@Cu shows an amorphous material (Figure 2). Analysis of PXRD samples using the JADE software program reveals that the final sorbed products of Cu^2+^ could be a mixture of compounds such as Cu^2+^_2_Cl (OH)_3_, Dy (OH)_3_, JCPDS PDF No. 19–043.0, JCPDS PDF No. 25-0269, and Cu_1.96_S, JCPDS PDF No. 29-0578, while the Pb^2+^ sorbed sample could be mainly made up of Pb (OH) Cl, JCPDS PDF No. 52-0289, and small amounts of C_4_H_4_O_4_PbS, JCPDS PDF No. 31-0694, Dy (OH)_3_, and JCPDS PDF No. 19–043.0 (Tables [Supplementary-material supplementary-material-1] and [Supplementary-material supplementary-material-1]).

### 3.2. Heavy Metal Detection Experiments

The synthesized tablets were used in heavy metal sorption experiments, and their results are recorded in Table 1. Dy_2_(OH)_5_(C_28_H_20_O_6_S_2_)_0.5_.nH_2_O (abbreviated as Dy-Stb) exhibited the best heavy metal sorption ability (Table 1). And, the sorption ability was mainly attributed to its high BET surface area and higher pore size and volume as compared to the other lanthanide-containing LDHs (Table 1 and [Supplementary-material supplementary-material-1]). Secondly, selective phosphorescence turn off/on for various heavy metal ions was investigated (Figure 1). Due to clear, distinct physical colour changes of the resultant Dy-Stb@Pb^2+^ and Dy-Stb@Cu^2+^ materials ([Supplementary-material supplementary-material-1]), they were studied further for possible field experiments in environmental point source pollutant monitoring experiments.

### 3.3. Phosphorescence Turnoff

**Figure 4:**
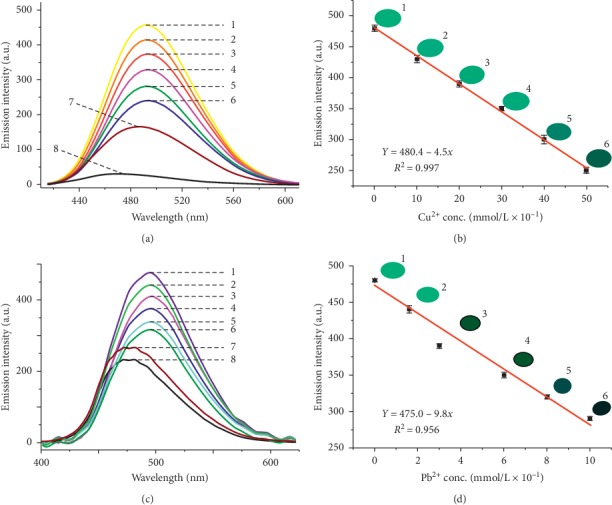
(a) Reducing emission intensities of Dy_2_(OH)_5_(C_28_H_20_O_6_S_2_)_0.5-_@Cu.nH_2_O tablets at different concentrations of Cu^2+^ ions and (b) the corresponding linear relationships; (c) reducing emission intensities of Dy_2_(OH)_5_(C_28_H_20_O_6_S_2_)_0.5−_@Pb.nH_2_O tablets at different concentrations of Pb^2+^ ions and (d) the corresponding linear relationships. Excitation wavelength = 365 nm, exit slit = 1 nm, PMT voltage = 700 v, adsorbent = (Dy_2_(OH)_5_(C_28_H_20_O_6_S_2_)_0.5_.nH_2_O), tablet weight = 1 g, and time of sorption = 45 minutes, and the tablets were placed in a running water experiment with a flow rate of 100 ml/s.

Phosphorescence measurements of the sorbed materials clearly varied in intensity from one concentration to another allowing the detection of various concentrations of the samples from the tablet sorption abilities (Figure 4). These results indicate that when the tablets are placed in the column water experiments, they sorb the heavy metal elements and the phosphorescence of stilbene ligand is turned off. The turnoff rate depends on the concentration of the water sample in the column, and by comparing the phosphorescence intensity of the original Dy-Stb sample to the sorbed samples, we are able to determine the concentration of unknown samples using the generated equations (Figure 4).

**Figure 5:**
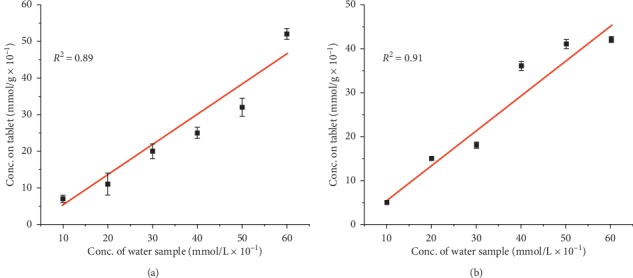
Concentrations of (a) Pb^2+^ and (b) Cu^2+^ ions sorbed by Dy_2_(OH)_5_(C_28_H_20_O_6_S_2_)_0.5_.nH_2_O tablets as determined from their digested samples by ICP. Note that using phosphorescence turnoff technique gives more accurate results (*R*^2^ = 0.96) than ICP measurements.

The effectiveness of determining the concentrations of the sorbed tablets using ICP is low as compared with using the phosphorescence turnoff (Figures 4 and 5). This is exhibited by the low *R*^2^ values recorded for ICP measurements of 0.89 and 0.91 for Pb^2+^ and Cu^2+^, respectively (Figure 4). This is in comparison with high *R*^2^ values of 0.96 and 0.99 for Pb^2+^ and Cu^2+^, respectively, in phosphorescence turnoff measurements (Figure 4). Therefore, it is noted that although direct heavy metal determination using ICP could give an indication of the pollutant concentration, better and accurate results can be found by determining the phosphorescence turnoff rate (Figure 4).

### 3.4. Photobleaching

The possibility of photobleaching of the Dy-Stb tablets in the field was tested by subjecting the tablets to intense UV irradiation for 72 hours ([Supplementary-material supplementary-material-1]). The results indicate the Dy-Stb sample to be extremely stable with a photobleaching rate of 2.4a.uh^−1^ as compared to stilbene which had a rate of 23a.uh^−1^. The stability of Dy-Stb against photobleaching is attributed to the antenna effect created by the lanthanide anions in Dy-Stb material ([Supplementary-material supplementary-material-1]) [11]. Whereby, stilbene absorbs light and reaches its singlet excited state before the energy is transferred to a triplet state with a relatively long lifetime. The energy is then transferred onto Dy^3+^ excited states. Thereafter, there occurs an internal conversion leading to an emissive state that is Dy-centred luminescence. Therefore, since absorption is stilbene centred while emission is Dy-centred, there is a wavelength red shift from 475 nm for stilbene emission to 500 nm for Dy-Stb emission ([Supplementary-material supplementary-material-1]). This phenomenon is called ligand-induced Stokes' shift (or Richardson's shift) and is responsible for the stability of the Dy-Stb tablets against photobleaching [10]. However, it is noted that the mechanisms of stilbene phosphorescence turnoff by metals should be studied further.

Furthermore, Dy-Stb material was stable up to 400°C ([Supplementary-material supplementary-material-1]). The intercalation of stilbene within the interlayer space of the LDHs prevents it from decomposition, hence its stability. As such, it may be possible to apply this material even in high-temperature sorption experiments. The sorption of the heavy metals was also found to be unaffected by pH values between 4 and 8 ([Supplementary-material supplementary-material-1]). Below and above these pH values, the Dy-Stb material dissociated in the solution. In addition, there was no ion exchange of stilbene in Dy-Stb starting material with any other competing ions in the solution such as halides. This was determined by titrimetrically checking halide concentrations after the Dy-Stb tablets were placed in their solutions for 45 minutes.

### 3.5. Leaching Capacity of the Intercalated Stilbene

The leaching rate of the Dy-Stb tablets placed in the column experiments was also determined from weight differences of original tablets and tablets placed in column experiments with pure distilled water. This rate was determined to be 0.01%, and it had a negligible effect on phosphorescence intensity. However, for ICP measurements, the weight loss is significant and a correction weight of 0.999 g (for original tablet weight) was used in calculating the reported values.

**Figure 6:**
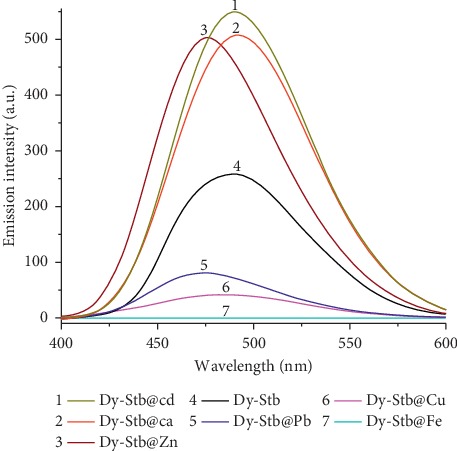
Luminescence emission intensities of different adsorbents: Dy-Stb = Dy_2_(OH)_5_(C_28_H_20_O_6_S_2_)_0.5_.nH_2_O.

In an environmental system containing multiple cations, phosphorescence measurement technique is less effective as each cation has a different turn on/off effect on the Dy-Stb sample (Figure 6). In such a case, direct determination of the sample cation concentration using ICP measurements is more preferred.

**Figure 7:**
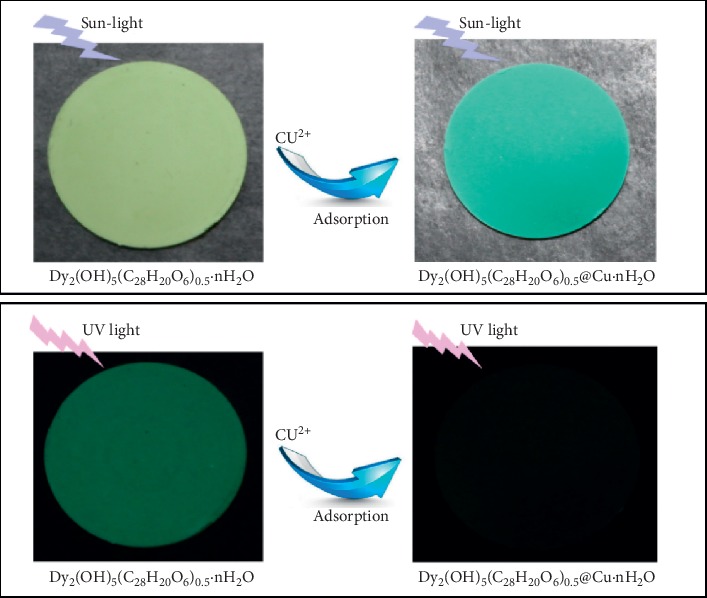
Phosphorescence turnoff of tablets made from layered rare-earth hydroxides intercalated with 4, 4-bis (2-sulfonatostyryl) biphenyl ([C_28_H_20_O_6_S_2_]^2−^) ions.

This robust environmental tool (Figure 7) might be helpful in effective monitoring procedures of point source activities and wastewater decontamination before discharge into aquatic ecosystems. The results for Pb^2+^ ions detected compares well with those obtained from facile fluorescence “turn on” sensing of lead ions in water via carbon nanodots immobilized in spherical polyelectrolyte brushes of 22.8 *μ*M [24]. The difference being that in our case, absorption of the metal ions results in phosphorescence turnoff. However, both techniques result in trace level detection mechanisms. De-Acha et al. [34] have summarized recent advances in phosphorescence sensors, and among the reported sensors, lead and copper ions have been detected in the range of 0 to 1 × 10^−5^ M. Detection of the current method is within a similar range of 0 to 1 × 10^−6^ M.

## 4. Conclusions

The anions of 4, 4′-bis (2-sulfonatostyryl) biphenyl ([C_28_H_20_O_6_S_2_]^2−^) are successfully intercalated within gallery spaces of lanthanide double hydroxide nanocomposites and characterized. The synthesized nanocomposites are used in making tablets that are used in absorption of heavy metal cations from aquatic ecosystems, and the contaminant concentration was determined. The detection mechanisms of trace metal cation result from fluorescence turn off/on of the synthesized nanocomposites. The concentrations of the cations are determined from their ability to turn off the phosphorescence of the stilbene intercalated LDHs tablets. And, the tablets were found to be stable against photobleaching.

## Figures and Tables

**Table 1:** Sorption of Pb^2+^ and Cu^2+^ with different stilbene intercalated lanthanide-containing layered double hydroxides and their physicochemical parameters.

Stilbene intercalated lanthanide-containing LDH material	BET SA (m^2^/g)	Langmuir SA (m^2^/g)	Pore vol. (cm^3^/g)	Pore size (nm)	Max. adsorp.^*∗*^ Pb^2+^ (mmol/g)	Max. adsorp.^*∗*^ Cu^2+^ (mmol/g)
Dy_2_(OH)_5_(C_28_H_20_O_6_S_2_)_0.5_.nH_2_O	32.111	450.965	0.065	8.154	5.672 ± 0.21	6.133 ± 0.01
Gd_2_(OH)_5_(C_28_H_20_O_6_S_2_)_0.5_.nH_2_O	31.475	189.204	0.062	7.872	3.23 ± 0.11	3.678 ± 0.05
Nd_2_(OH)_5_(C_28_H_20_O_6_S_2_)_0.5_.nH_2_O	28.233	102.596	0.085	12.065	2.612 ± 0.32	3.089 ± 0.09
Sm_2_(OH)_5_(C_28_H_20_O_6_S_2_)_0.5_.nH_2_O	22.276	92.444	0.039	7.086	2.143 ± 0.25	2.321 ± 0.06
Yb_2_(OH)_5_(C_28_H_20_O_6_S_2_)_0.5_.nH_2_O	10.134	62.507	0.030	11.938	1.872 ± 0.12	2.043 ± 0.04
Er_2_(OH)_5_(C_28_H_20_O_6_S_2_)_0.5_.nH_2_O	2.249	32.261	0.012	21.968	0.987 ± 0.34	1.542 ± 0.02
Tb_2_(OH)_5_(C_28_H_20_O_6_S_2_)_0.5_.nH_2_O	11.356	12.327	0.040	14.134	0.541 ± 0.55	1.123 ± 0.10
Eu_2_(OH)_5_(C_28_H_20_O_6_S_2_)_0.5_.nH_2_O	1.878	2.959	0.005	10.464	0.523 ± 0.12	0.341 ± 0.02

^*∗*^Maximum adsorption time = 45 minutes; solution pH = 4–8; Temperature = 298 K; SA = Surface area; vol. = Volume

## Data Availability

All the necessary information required for replication of this work and/or conducting secondary analysis is included within the article.

## Supplementary Materials

Supplementary Materials Analysis of PXRD using the JADE program, and images of tablets, BET, TGA, pH, and photobleaching graphs: Table S1. XRD characterization of Dy2(OH)5(C28H20O6S2)0.5@Pb.nH2O composite material. Table S2. XRD characterization of Dy2(OH)5(C28H20O6S2)0.5@Cu.nH2O composite material. Figure S1. BET for Dy2(OH)5(C28H20O6S2)0.5.nH2O. Figure S3. (a) stilbene and (b) Dy2(OH)5(C28H20O6S2)0.5.nH2O photobleaching experiments. Proposed mechanisms that help to prevent photobleaching of stilbene intercalated into a lanthanide-containing layered double hydroxide material (antenna effect). Abbreviations: *A* = absorption; *F* = fluorescence; *P* = phosphorescence; *L* = lanthanide-centred luminescence; ISC = intersystem crossing; ET = energy transfer; *S* = singlet; *T* = triplet. Full vertical lines indicate radiative transitions; dotted vertical lines indicate nonradiative transitions. Figure S5. TGA for Dy2(OH)5(C28H20O6S2)0.5.nH2O. Figure S6. Effect of pH on adsorption ability of Dy2(OH)5(C28H20O6S2)0.5.nH2O tablets.
