# Supplementary material for: Trace Metal Detection in Aqueous Reservoirs Using Stilbene Intercalated Layered Rare-Earth Hydroxide Tablets
Source: J Anal Methods Chem. 2020 Apr 12;2020:9712872. doi: 10.1155/2020/9712872 (PMC7152999; doi:10.1155/2020/9712872)
Supplement: Supplementary Materials — Analysis of PXRD using the JADE program, and images of tablets, BET, TGA, pH, and photobleaching graphs: Table S1. XRD characterization of Dy2(OH)5(C28H20O6S2)0.5@Pb.nH2O composite material. Table S2. XRD characterization of Dy2(OH)5(C28H20O6S2)0.5@Cu.nH2O composite material. Figure S1. BET for Dy2(OH)5(C28H20O6S2)0.5.nH2O. Figure S3. (a) stilbene and (b) Dy2(OH)5(C28H20O6S2)0.5.nH2O photobleaching experiments. Proposed mechanisms that help to prevent photobleaching of stilbene intercalated into a lanthanide-containing layered double hydroxide material (antenna effect). Abbreviations: A = absorption; F = fluorescence; P = phosphorescence; L = lanthanide-centred luminescence; ISC = intersystem crossing; ET = energy transfer; S = singlet; T = triplet. Full vertical lines indicate radiative transitions; dotted vertical lines indicate nonradiative transitions. Figure S5. TGA for Dy2(OH)5(C28H20O6S2)0.5.nH2O. Figure S6. Effect of pH on adsorption ability of Dy2(OH)5(C28H20O6S2)0.5.nH2O tablets. [file 9712872.f1.doc]

Trace metal detection in aqueous reservoirs using stilbane intercalated layered rare earth hydroxide tablets

Solomon Omwoma

Jaramogi Oginga Odinga University of Science and Technology,

Box 210-40601, Bondo, Kenya.

**Supporting Information**

**Table S1**. XRD Characterization of Dy_2_(OH)_5_(C_28_H_20_O_6_S_2_)_0.5_@Pb.nH_2_O composite material

| **Peak No.** | **2-Theta** | **d(nm)** | **hkl** | **Phase ID** |
| --- | --- | --- | --- | --- |
| 1 | 15.418 | 0.57424 | (101) | Pb(OH)Cl |
| 2 | 18.332 | 0.48355 | (002) | Pb(OH)Cl; C_4_H_4_O_4_PbS (0.215) |
| 3 | 22.217 | 0.39981 | (102) | Pb(OH)Cl |
| 4 | 23.979 | 0.37081 | (011) | Pb(OH)Cl; C_4_H_4_O_4_PbS (-0.013) |
| 5 | 25.027 | 0.35551 | (200) | Pb(OH)Cl; C_4_H_4_O_4_PbS (0.254) |
| 6 | 27.079 | 0.32901 | (111) | Pb(OH)Cl; C_4_H_4_O_4_PbS (-0.082) |
| 7 | 31.553 | 0.28331 | (112) | Pb(OH)Cl |
| 9 | 34.923 | 0.2567 | (211) | Pb(OH)Cl; C_4_H_4_O_4_PbS (0.241) |
| 10 | 35.617 | 0.25186 | (013) | Pb(OH)Cl |
| 11 | 37.059 | 0.24239 | (004) | Pb(OH)Cl |
| 12 | 37.56 | 0.23926 | (203) | Pb(OH)Cl; C_4_H_4_O_4_PbS (-0.120) |
| 13 | 39.161 | 0.22985 | (104) | Pb(OH)Cl |
| 14 | 42.472 | 0.21266 | (302) | Pb(OH)Cl |
| 15 | 44.021 | 0.20553 | (213) | Pb(OH)Cl; C_4_H_4_O_4_PbS (0.120); Dy(OH)_3_ (-0.106) |
| 16 | 45.086 | 0.20092 | (311) | Pb(OH)Cl; C_4_H_4_O_4_PbS (-0.254) |
| 17 | 48.343 | 0.18812 | (122) | Pb(OH)Cl; C_4_H_4_O_4_PbS (0.065) |
| 18 | 50.802 | 0.17957 | (122) | Pb(OH)Cl |
| 19 | 52.299 | 0.17478 | (015) | Pb(OH)Cl; C_4_H_4_O_4_PbS (-0.071) |
| 20 | 53.962 | 0.16978 | (115) | Pb(OH)Cl; Dy(OH)_3_ (0.061) |
| 21 | 56.637 | 0.16238 | (410) | Pb(OH)Cl |
| 22 | 59.12 | 0.15614 | (124) | Pb(OH)Cl |
| 23 | 61.24 | 0.15123 | (206) |  |
| 24 | 63.677 | 0.14602 | (322) | Pb(OH)Cl |
| 26 | 66.401 | 0.14067 | (322) | Pb(OH)Cl |
| Pb(OH)Cl, JCPDS PDF No. 52-0289; C_4_H_4_O_4_PbS, JCPDS PDF No. 31-0694; Dy(OH)_3_, JCPDS PDF No. 19-0430; The value in brackets indicates the deviation of 2 Theta values from the indicated phase IDs | | | | |

**Table S2**. XRD Characterization of Dy_2_(OH)_5_(C_28_H_20_O_6_S_2_)_0.5_@Cu.nH_2_O composite material

| Peak No. | 2-Theta | d(nm) | ( h k l ) | Phase ID |
| --- | --- | --- | --- | --- |
| 1 | 16.328 | 0.54064 | ( 0 1 1) | Cu^2+^_2_Cl(OH)_3_ (-0.102) |
| 3 | 32.563 | 0.27475 | ( 1 0 3) | Cu_1.96_S (0.011) |
| 5 | 32.865 | 0.27229 | ( 2 0 0) | Dy(OH)_3_ (0.036) |
| 6 | 39.955 | 0.22546 | ( 2 0 2) | Cu^2+^_2_Cl(OH)_3_ (-0.071) |
| 7 | 50.091 | 0.18195 | ( 2 0 3) | Cu^2+^_2_Cl(OH)_3_ (-0.011) |
| 8 | 53.633 | 0.17074 | ( 2 1 2) | Cu_1.96_S (0.037) |
| The value in brackets indicates the deviation of 2 Theta values from the indicated phase IDs; Cu^2+^_2_Cl(OH)_3_, JCPDS PDF No. 25-0269; Cu_1.96_S, JCPDS PDF No. 29-0578. | | | | |

**Fig. S1.** BET for Dy_2_(OH)_5_(C_28_H_20_O_6_S_2_)_0.5_.nH_2_O

**Fig S2**. Clear and distinct colour changes resulting from a) Pb^2+^ and b) Cu^2+^ ions sorption by Dy_2_(OH)_5_(C_28_H_20_O_6_S_2_)_0.5_.nH_2_O tablets.

**Fig. S3**. a) Stilbane, b) Dy_2_(OH)_5_(C_28_H_20_O_6_S_2_)_0.5_.nH_2_O photo-bleaching experiments.

**Fig. S4**. Proposed mechanisms that help to prevent photo-bleaching of stilbane intercalated into a lanthanide containing layered double hydroxide material (antenna effect). Abbreviations: A = Absorption; F = fluorescence; P = phosphorescence; L = lanthanide-cantered luminescence; ISC = intersystem crossing; ET = energy transfer; S = singlet; T = triplet. Full vertical lines indicate radiative transitions; dotted vertical lines indicate non-radiative transitions.

**Fig. S5**. TGA for Dy_2_(OH)_5_(C_28_H_20_O_6_S_2_)_0.5_.nH_2_O

**Fig. S6.** Effect of pH on adsorption ability of Dy_2_(OH)_5_(C_28_H_20_O_6_S_2_)_0.5_.nH_2_O tablets.

**Instruments**

Measurements of diffraction patterns were achieved using a Rigaku XRD diffractometer machine (6000) conditioned at 30 mA, 40 kV, Cu-Ka (λ = 0.154 nm and with a scan step of 0.01° measured between 3° - 70°. Surface morphology was observed using a SEM (Zeiss Supra 55) machine simultaneously connected to an EDX detector. Cross sectional transverse morphological study was done using a TEM machine (Hitachi H-800). Emission fluorescence was measured using a Hitachi F-7000 spectrometer with a Xe lamp as the excitation source. High sensitivity elemental analysis in aqueous solutions was performed using an ICP-AES machine (ICPS-7500). Solid samples were first digested using Aqua ragia solutions before ICP-AES analysis. Thermogravimetric stability was determined using a locally-produced HCT thermal analysis system in N_2_ environment at a heating rate of 10 ^o^C min^-1^. The oxidation status of specific metal elements in the synthesized materials was determined using an X-ray photoelectron spectroscopy (XPS) machine with monochromatized Al Kα exciting X-radiation (PHI Quantera SXM). The pore volumes and specific surface area studies for the solid sample were done using BET and BJH methods with Quanta-chrome Autosorb1C VP machine. Before such analysis was done, the solid samples were first degassed @ 100 ^o^C for 6 hrs. The OH content of solid samples was determined titrimetrically by neutralization back-titration after the dissolution of the samples in a 0.1 N standard H_2_SO_4_.
